# Successful Pediatric Mechanical Thrombectomy for HeartMate 3-Related Intracranial Thromboembolism

**DOI:** 10.1177/21501351221075840

**Published:** 2022-03-03

**Authors:** Catherine Proulx, Alejandro Floh, Aamir Jeewa, Elizabeth Pulcine, Prakash Muthusami, Leonardo R. Brandão, Osami Honjo, Carolina Vargas, Andrea Maurich, Mjaye Mazwi

**Affiliations:** 1 Department of Critical Care Medicine, 7979 The Hospital for Sick Children, Toronto, Canada; 2 Division of Cardiology, 7979 The Hospital for Sick Children, Toronto, Canada; 3 Division of Neurology, 7979 The Hospital for Sick Children, Toronto, Canada; 4 Department of Diagnostic Imaging, 7979 The Hospital for Sick Children, Toronto, Canada; 5 Division of Haematology/Oncology, 7979 The Hospital for Sick Children, Toronto, Canada; 6 Division of Cardiovascular Surgery, 7979 The Hospital for Sick Children, Toronto, Canada; 7 Department of Paediatrics, 12366 University of Toronto, Toronto, Canada; 8 Department of Surgery, 12366 University of Toronto, Toronto, Canada

## Abstract

Thromboembolic events post left ventricular assist devices (LVAD) implantation remain a major cause of morbidity and mortality. Mechanical thrombectomy for the treatment of pediatric intracranial thromboembolic events have been reported in LVADs, but never following HeartMate 3 (HM3) implantation. We present the case of an 8-year-old, 26.5 kg male with dilated cardiomyopathy and decompensated heart failure who presented with extensive intracranial thromboembolism in the early postoperative period following HM3 implantation and underwent successful mechanical thrombectomy with a favorable neurological outcome.

## Background

Despite improved survival post left ventricular assist device (LVAD) implantation, stroke remains a major cause of morbidity and mortality and can limit transplant eligibility.^1^ Centrifugal-flow LVADs such as the HeartMate 3 (HM3) have a reduced stroke risk.^2^ Mechanical thrombectomy has been reported for the treatment of intracranial thromboembolic events following LVAD implantation,^3^ including in children,^4^ but has not been reported in a pediatric patient following HM3 implantation. We report a successful mechanical thrombectomy for an extensive intracranial thromboembolism post HM3 implant in a child.

## Case Presentation

An 8-year-old, 26.5 kg male (body surface area 0.93 m^2^) with dilated cardiomyopathy (*MYBPC3* mutation) and decompensated heart failure underwent uncomplicated HM3 implantation as a bridge to cardiac transplantation. Red blood cells and platelets were transfused during the operative procedure. Hemostasis was satisfactory, and there was no coagulopathy. Intraoperative transesophageal echocardiography did not identify any intracardiac communication or thrombi. In the immediate postoperative period, HM3 flows were below 2 L/min (indexed flows of 2.2 L/min/m^2^ at 5200 RPM) due to marginal right ventricular function, with a 1 h period down to a low of 1.4 L/min 12 h, postoperatively. Supportive measures included afterload reduction, epinephrin, pacing, and sedation. Despite low flows, blood pressure and markers of cardiac output remained adequate. There was no significant postoperative coagulation derangement.

**Figure 1.**
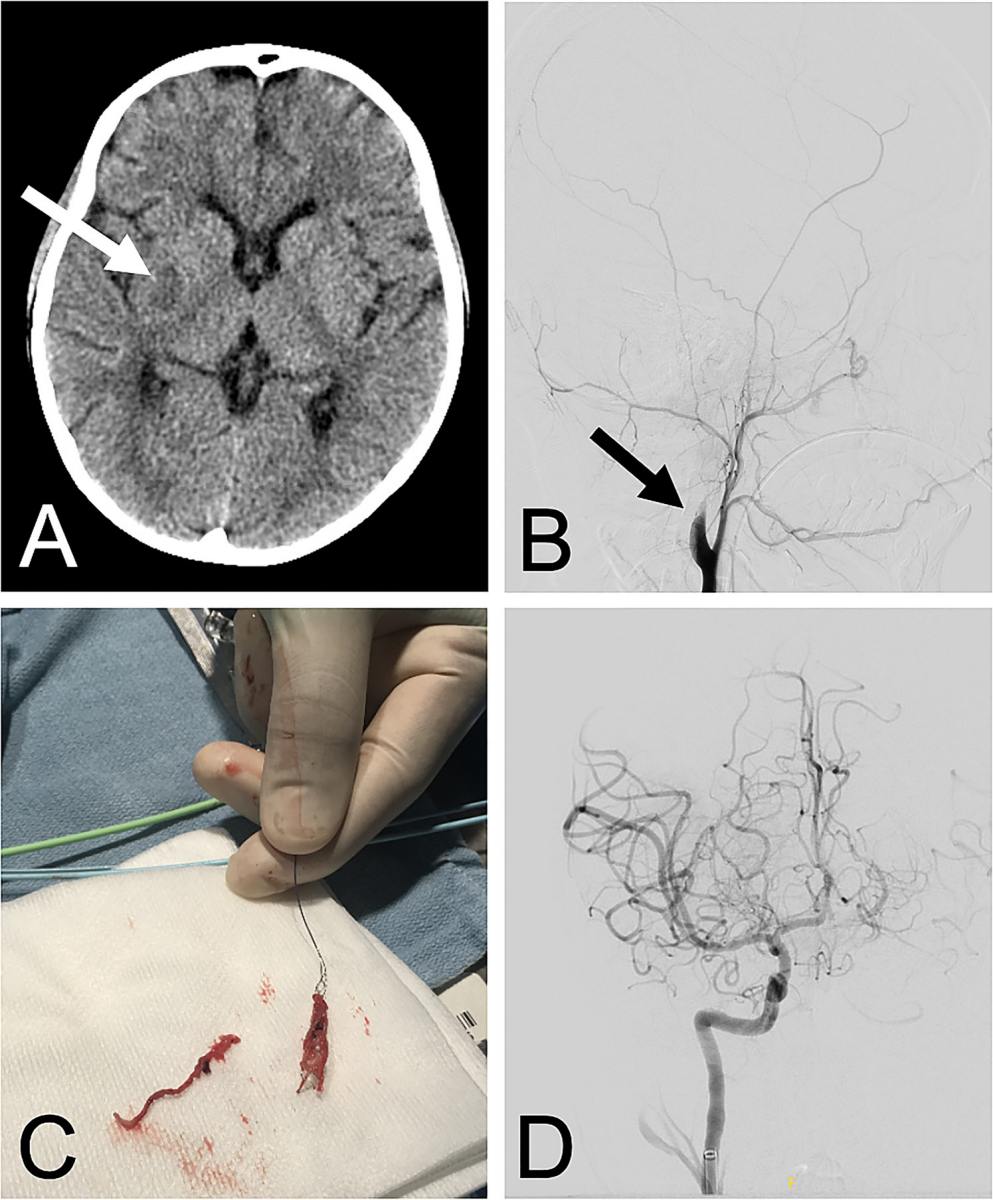
(A) Computed tomography with angiography showing an established right basal ganglia infarct; (B) initial digital subtraction angiography demonstrating absence of flow distal to the right carotid bifurcation; (C) stent retriever with a fresh clot embedded and a second tubular thrombus laying on the side; (D) post thrombectomy digital subtraction angiography confirming complete recanalization of the occluded circulation (color images available in online version).

Eighteen hours after surgery and 1 h after initiation of anticoagulation with unfractionated heparin (UFH), sedation was lifted in anticipation of tracheal extubation. As the patient emerged from sedation, a dense left hemiparesis was recognized (Pediatric National Institute of Health Stroke Scale of 17 while intubated). Given the risk of stroke, UFH was held, and urgent computed tomography with angiography (CT/A) was performed. Neuroimaging revealed an established right basal ganglia infarct (Figure 1A) with a large vessel occlusion arising from the right common carotid bifurcation and extending the length of the right internal carotid artery to the proximal middle cerebral artery. No intracardiac or pump thrombus was found on cardiac CT/A.

A consensus decision was made for urgent endovascular thrombectomy to prevent infarct extension. Thrombectomy was initiated 2 h after hemiparesis recognition. Digital subtraction angiography confirmed no flow distal to the right carotid bifurcation (Figure 1B). Six transfemoral endovascular passes via Solumbra (use of stent retriever in combination with aspiration) and ADAPT (A direct Aspiration First Pass Technique) achieved complete recanalization of occluded arteries (Figure 1D) (Modified Thrombolysis in Cerebral Infarction Scale of 3). Follow-up serial head CTs did not show evidence of reperfusion injury, intracranial hemorrhage, or cortical infarct extension. Therapeutic anticoagulation with UFH was commenced 6 h post mechanical thrombectomy after a CT confirming lack of hemorrhagic conversion. Improvement of his left-sided hemiparesis was observed 36 h from symptom onset. He was uneventfully extubated on postoperative day (POD) 4 of the HM3 implantation. Aspirin was started on POD 5 and warfarin on POD 6 for an international normalized ratio target of 2 to 3. He was transferred to the cardiology ward on POD 9. Intensive rehabilitation was undertaken to optimize motor outcome.

At 3 months follow-up, the patient demonstrates ongoing neurological improvement with inpatient rehabilitation therapy. His Pediatric Stroke Outcome Measure is 1. He scores 0.5 for both a left sensorimotor and a cognitive/behavioral deficit, which correspond to mild impairments with no impact on function. He ambulates autonomously, can throw a ball, feeds orally and converses normally. Given the basal ganglia involvement, he developed a postural dystonic tremor and a left upper extremity hyperesthesia which have improved with clonazepam and gabapentin therapy, respectively.

## Comment

When ischemic strokes are associated with LVADs, decision making around anticoagulation is fraught, requiring weighing the risks of device thrombosis versus hemorrhagic conversion of an infarct or systemic hemorrhage, particularly in the setting of large-territory infarcts. In adults with ischemic strokes and LVADs, thrombolysis is generally contraindicated given the risk of hemorrhage, but thrombectomy is an option in eligible patients.^3^ In adults with ischemic stroke due to large vessel occlusion, thrombectomy, with or without thrombolysis, is standard-of-care and associated with superior neurological outcomes compared to conservative therapy.^5^ To date, no guidelines supported by randomized controlled trials in pediatric stroke patients exist.

Pediatric evidence suggests that mechanical thrombectomy can be safe and feasible in large vessel occlusion stroke,^6^ including in children with LVADs when carefully selected by pediatric stroke neurologists and interventional radiologists.^4^ Pediatric guidelines for both patient selection and optimal thrombectomy technique are required to maximize benefits and minimize risks. In the interim, risk assessment and decision-making needs be individualized. To our knowledge, this is the first reported case of pediatric mechanical thrombectomy after HM3 implantation.

In this patient, exact timing of stroke is unknown but suspected to have occurred during the period of low LVAD flow. The small infarct with such extensive thrombus suggested acute onset (<24 h prior to diagnosis). Our decision to undertake mechanical thrombectomy was guided by (i) the acute nature of the extensive thrombus, (ii) a large penumbra with mismatch between clinical deficit and established small infarct, and (iii) expected progression to extensive cerebral infarction and malignant middle cerebral artery syndrome if left untreated. Multidisciplinary collaboration was key to deciding timing of neuroimaging, initiation, and escalation of anticoagulation, and choice of the most appropriate thrombectomy techniques.

## Conclusion

Close neurological monitoring post LVAD implantation is key for timely diagnosis and treatment of stroke. Given the absence of pediatric stroke guidelines, a multidisciplinary approach is imperative. Successful recanalization of a large vascular territory with subsequent neurological recovery without procedure-related complications in our patient supports the possibility of mechanical endovascular thrombectomy in the setting of acute large vessel thromboembolic events in suitable pediatric patients with LVADs.
